# A simulation study on the process design and optimization pressure swing separation of azeotropic mixture methanol and toluene

**DOI:** 10.1371/journal.pone.0310541

**Published:** 2024-12-23

**Authors:** Xinxin Liu, Ndungutse Jean Maurice, Mugabekazi Joie Claire, Bigirimana Gentil, Junning Li, Zengxiang Jiao, Abdulmoseen Segun Giwa

**Affiliations:** 1 Nanchang Institute of Technology, The School of Hydraulic & Ecological Engineering, Nanchang, China; 2 Institute of Environmental Science, Shanxi University, Taiyuan, China; 3 Faculty of Education, Southwest University, Chongqing, China; 4 College of Architecture and Civil Engineering, Xi’an University of Science and Technology, Xi’an, China; 5 Nanchang Institute of Science and Technology, School of Civil and Environmental Engineering, Nanchang, China; University of New Hampshire, UNITED STATES OF AMERICA

## Abstract

Pressure Swing Distillation (PSD) is the only advanced technology that does not require the addition of third components to the system to enhance the separation of azeotropic mixtures. It outperforms homogeneous distillation for separating pressure-sensitive azeotropic mixtures. In this study, we aimed to separate methanol and toluene using the Non-Random Two-Liquid (NRTL) and Aspen Plus thermodynamic calculation models to simulate a binary homogeneous azeotropic system. The standard PSD process was employed to separate methanol and toluene. Furthermore, multiple optimization sequences were utilized to sequentially optimize the process for obtaining higher purities of methanol and toluene while reducing the Total Annual Cost (TAC) and heat energy consumption. The effects of the optimization sequence on the TAC were investigated. The best optimization sequences for graphing in Origin or Aspen Plus were found to be RR1, NR, NF1, NF2, NT1, and NT2. Additionally, the Double-Effect Distillation (DED) optimization sequence is similar, with TAC as the primary function in the simulation and methanol and toluene purities up to 99.99%. In the DED simulation, the feed position and tray number were found to be sensitive to TAC by the order NR > NF1 > NF2 and NT1 > NT2. This study simulated PSD using the NRTL thermodynamic calculation model in Aspen Plus and generated visualizations using Origin software.

## Introduction

Methanol and toluene have emerged as pivotal organic solvents and essential chemical feedstocks in the pharmaceutical and fine chemical industries due to their extensive application potential. Methanol, a fundamental basic chemical raw material, also serves as a superior-quality fuel with widespread utilization in various aspects of daily life [1]. The high solvent capacity of both compounds renders them valuable for a multitude of applications across different fields. They are prevalently used as solvents in the synthesis of dyes, coatings, inks, alkaloids, paints, and adhesives, and are particularly vital in formulating novel high-performance special epoxy resin intermediates. As solvents, they are compatible with a range of organic compounds, including ethers, benzenes, and ketones, and serve as auxiliary additives to enhance the octane number. Furthermore, methanol and toluene are extensively applied as cleaning agents across diverse sectors such as medical, light industry, transportation, textiles, and electronics. Additionally, their usage in industrial processes results in a significant volume of organic waste liquids within the chemical industry. The process of methanol and toluene alkylation, particularly for the production of the key aromatic compound para-xylene under the influence of improved catalysts, presents substantial prospects for development [2]. In pharmaceutical manufacturing, methanol and toluene are involved in hydrogenation procedures to produce m-xylene; this application generates considerable quantities of methanol/toluene waste liquids, as seen in the production of the medication Naofukang (pyracetamide).

Under atmospheric pressure, the waste liquid forms an azeotropic mixture with 88.20 mol% methanol and 11.80 mol% toluene, boasting an azeotropic temperature of 63.8°C [3]. The inefficiency of conventional rectification methods to separate this azeotrope complicates the recycling process. The adoption of pervaporation using an organosilica membrane as a strategy to recover and recycle methanol/organic azeotropes presents an alternative approach [4]. Nevertheless, the complexity of this technique, coupled with high operational costs and the challenges associated with scaling up for industrial use, limits its applicability. In addition, the necessity of introducing a third component to facilitate the process further increases the cost of treatment and introduces the risk of product contamination.

Other studies also reported the methanol and toluene separation conundrum techniques. S. Moulik et al. [5] utilized chitosan-polytetrafluoroethylene composite membrane. The diffusion coefficients of methanol and toluene were found to be 1.7 × 10^−9^ and 1.8 × 10^−12^ m^2^/s, respectively. The principles of batch extraction and rectification, using o-xylene as the extractant to yield a methanol product with a mole fraction of 99.688% was achievable [6]. Additionally, yields of over 95% for toluene and more than 93% for methanol were reported, exemplifying the effectiveness of the separation technique employed [2].The methods outlined in previous studies have demonstrated effective separation of the system. However, only a limited number of theoretical studies have investigated the separation of methanol/toluene using PSD. Traditional rectification processes require a higher reflux ratio and more theoretical plates, leading to increased distillation energy consumption and equipment costs, which constrain its application. In contrast, the PSD process does not require the introduction of a third component, as is the case with extractive distillation. Instead, it utilizes pressure to separate compounds with close boiling points by significantly altering their relative volatility. The necessity for third components in processes such as extractive distillation increases the costs associated with waste liquid treatment and may potentially contaminate the product, thereby complicating recycling efforts. For instance, Weifeng Shen et al. [7] noted that a third solvent might interact more effectively with one component (either toluene or methanol), modifying its volatility characteristics and facilitating its retention in the liquid phase during evaporation, thus enhancing its separation from the other substance. If the chosen additive forms an azeotrope with either methanol or toluene, effective separation can occur by substantially lowering their volatilities beyond what could be achieved with a simple rectification or distillation unit without first breaking the azeotrope. Moreover, the liquid-liquid extraction method can utilize another immiscible, non-volatile organic solvent, such as water, which preferentially favors one component over the other [8]. This indicates that when water is added, methanol, being polar, mixes more readily, leaving behind less soluble organic compounds like toluene, thus facilitating easier separation. Seri Maulina et al. [9] reported that the adsorption process can be employed using various adsorbent materials, such as zeolites or activated charcoal, which preferentially adsorb or separate desired compounds based on differential absorption affinity.

Different methods such as PSD, extractive distillation, azeotropic distillation, reactive distillation, catalytic distillation, and other unique separation technology are currently the most successful separation methods for binary homogenous azeotrope systems [10]. The PSD process is advantageous due to its simple operation, elimination of the need for a third component, high separation efficiency, lower capital investment, energy saving, easy to control and high purity products [11]. Xin et al. [12] studied PSD to separate ethanol/acetonitrile binary azeotrope. In order to save costs, heat pump technology was applied to the swing distillation process, around 62.8% of the total operating cost was saved by using heat pump to assist PSD. And two new swing distillation processes were proposed. Min et al. [13] studied an economical and stable three-column variable pressure distillation process for the separation of methyl ethyl ketone (MEK), isopropanol (IPA) and ethanol (EtOH) terazeotrope, which provided a new idea for the separation of MEK/IPA/EtOH. Zhaoyou et al. [14] proposed a method called three-tower swing distillation to separate and separate acetonitrile/methanol/benzene ternary azeotrope, and confirmed the feasibility of the method using residue graphs. In addition, Amina et al [15] examined mechanical and thermal properties of polystyrene-co-butadiene as pervaporation membrane to separate toluene and methanol.

The sequential modular approach in the optimization process of PSD is favored for its intuit ive nature and lower computer memory requirements, rendering it an optimization technique that engineers find relatively easy to adopt and is, consequently, widely used in practice [16]. Zhao et al. [17] proposed an energy-efficient liquid-liquid extraction combined with heterogeneous azeotropic distillation or extractive distillation process based on traditional two-column heterogeneous azeotropic distillation and three-column extractive distillation. The reductions of 40.24% in the TAC and 45.37% in CO_2_ emissions was achieved compared with the traditional two-column heterogeneous azeotropic distillation process. Therefore, the two processes are more attractive in terms of both economic and environmental protection. In order to further reduce process energy consumption, Zhu et al.[18] explored several energy-saving processes, and optimized the extractive distillation process based on sequential iterative optimization algorithm with the total annual cost as the objective function. Shan et al. [19], the acetonitrile/benzene/methanol ternary homogeneous azeotrope system. It developed PSD approach using sequential module techniques as the optimization model and compared two methods for distillation sequence optimization, with the minimum TAC as the objective function. Zhao et al.[20] employed a sequential iterative approach to optimize the tetrahydrofuran/methanol binary azeotrope system, aiming for the minimal TAC. This optimization process design for achieving the best steady-state involved using the feed plate position as an inner iteration cycle. Mishra et al. [21] explored the separation of a methanol and isopropyl acetate binary mixture into its pure components using distillation. It also examined the binary homogeneous azeotrope system of dimethyl carbonate and methanol, employing the reflux ratio as the primary inner circulation and the feed position as the secondary inner circulation, with TAC as the objective function. The sequential module’s optimal design for the outermost circulation hinges on the precise number of trays, leading to an optimized process. The optimization results indicate that employing a partial heat integration technique can lower the TAC by 20.01% compared to the conventional distillation technology. This study’s simulation and optimization efforts target the separation of the toluene and methanol binary homogeneous azeotrope system using the industrially established and mature PSD process. Additionally, this research has systematically explored the conventional separation methods and thermal integration simulation. This is based on both the recovery and utilization of the waste liquid from toluene and methanol and the governing principles of the PSD process. The expected findings of the study are set to provide a distinct reference point and value for the separation of binary homogeneous azeotropic systems. These findings aim to establish a foundation for industrial application, thereby enhancing the potential for azeotropic system separation to be adopted in industrial contexts.

Presently, researchers predominantly enhance the distillation process by optimizing the reflux ratio, subsequently the feed location, and ultimately the total number of trays [22]. Yet, studies have not examined the effects of varying the optimization sequence for the reflux ratios of the two towers, the feed placement, and the number of theoretical plates on the objective function. This research aims to assess the economic ramifications of differing optimization sequences on the PSD process’s efficiency, utilizing the methanol/toluene binary minimum azeotropic system to gauge the sensitivity of optimization variables to the TAC. Such optimization emerges as an innovative approach for energy conservation and consumption reduction, vital for decreasing energy utilization within the rectification process. The evolution of production technology has steered industrial production toward a technologically advanced, quality-focused trajectory amid the rapid growth of modern industry. While comprehensive energy utilization promotes societal development, it simultaneously contributes to significant environmental pollution and inefficient energy use in contemporary manufacturing procedures. Consequently, the energy challenge, a pivotal indicator of industrial productivity, has become an urgent issue requiring immediate attention. Distillation technology harbors significant research potential to address both the energy crisis and environmental pollution efficaciously.

## Materials and methods

### Model selection and physical property of the technique model

The accuracy of simulation analysis data relies heavily on the selection of an appropriate physical property approach, which may directly impact the precision of the simulation outcomes. The methanol/toluene system, characterized as a highly non-ideal solution, is based on the fundamental characteristics of the system and draws upon the basic simulation methodologies of similar systems [23]. Wilson and NRTL equation have roughly the same correlation and prediction accuracy, and can be applied to multi-component gas-liquid phase equilibrium systems and liquid-liquid phase stratified systems. Both equations are mathematical models used to predict the behavior of chemical mixtures. They estimate quantities like activity coefficients, which tell us how non-ideal a mixture is behaving. Both these methods involve complex calculations that consider factors such as temperature, pressure and composition.

Respectively developed by Grant M.Wilson in 1964 [24] and Renon & Prausnitz in 1968 [25]; the two equations incorporate different assumptions about molecular interactions but have been shown to provide comparably accurate predictions for many types of mixtures. Consequently, the model equation was selected for simulating the physical property data of the system, with the binary interaction parameters of the NRTL equation estimated using the group contribution method in this study. Given the highly non-ideal nature of the methanol-toluene system, Trofimova et al. [26] reported the gas-liquid equilibrium data for this system and utilized the physical data regression system in Aspen Plus to align the NRTL physical property model with the methanol-toluene gas-liquid equilibrium data. The fitting and regression outcomes demonstrate that the method for physical characteristics is in agreement with the experimental data, thereby enabling its application in describing the PSD process for the methanol-toluene system pair. Below is the equation and component of the equation used for NRTL model in the liquid-liquid equilibrium.


$$\ln\;\gamma_{i}=\sum_{j=1}^{n}\tau_{ji}G_{ji}x_{j}/\sum_{k=1}^{n}G_{ki}k_{x}+\sum_{j=1}^{n}(x_{i}G_{ij}/\sum_{k=1}^{n}G_{kj}x_{k})[\tau_{ij}-\sum_{k=1}^{n}x_{k}\tau_{ki}G_{kj}/\sum_{k=1}^{n}G_{kj}x_{k}]$$

Where γ-is the activity coefficient

X—The mole fraction

N—The number of components

$$G_{ij}=\exp\left(-c_{ij}\tau_{ij}\right)$$ (1)

$$\tau_{ij}=a_{ij}\frac{b_{ij}}{T}+e_{ij}\;\ln\;T+f_{ij}T$$ (2)

$$C_{ij}=\delta_{ij}+d_{ij}(T-273.154K)$$ (3)

$$\tau_{ij}=0$$ (4)

$$G_{\mathrm{ij}}=1$$ (5)

Pertaining to the saturated vapor pressure calculation of liquid one refers ourselves to the Antoine equation [27] which is:

$$\ln\;P_{i}^{*}=C_{\mathrm{li}}+\frac{C_{2i}}{T+C_{3i}}+C_{4i}T+C_{5i}\;\ln\;T+C_{6i}T^{C_{7i}}$$ (6)

With $C_{1i}C_{7i}$ being a parametric model for calculation.

### Economic cost of the model

TAC is a pivotal metric for assessing the cost-effectiveness of a procedure. It serves as an essential indicator of the economic viability of a new method, comprising two primary components: operating costs and equipment costs [28]. In this system, the equipment cost predominantly includes a rectification tower and a heat exchanger, with other minor equipment costs being negligible in comparison and thus can be disregarded. Conversely, the operating cost mainly encompasses public utility expenses. The TAC formulas, sourced from Luyben et al. [29], provide an economic accounting framework for simulating and optimizing technical processes with TAC as the objective function. This framework integrates both equipment and energy costs into the economic cost model. The energy consumption cost for the system accounts for the expenses associated with steam and cooling water, with the annual operation set at 8000 hours. The diameter of the tower is determined using the Tray Sizing function in the Aspen Plus software, with a default plate spacing of 0.61 meters. According to a simulation study by Qasim et al. [30], the comprehensive economic feasibility of the process is evaluated using objective functions such as project capital cost, total operating cost, and capital depreciation. A comparative analysis reveals that the extractive distillation process, when juxtaposed with PSD, appears more economical due to its lower electricity usage and potential for increased income through heat integration. Economic optimization models vary in their attributes. Additionally, this study amalgamates Luyben’s and Douglas’s economic models, specifically their approach to calculating cooling water costs, to enhance the process simulation optimization. Table 1A and 1B delineate the specific parameters used in economic accounting.

**Table 1:** a. Basis of economic accounting of the cost model: TAC. b. Basis of economic accounting of the cost model: Monitoring price.

**TAC = (device cost)/ (payback period) + energy cost**
**Device cost = Condenser cost + Re-boiler cost + Vessel cost**
**Condenser**
Coefficient of heat transfer = 0.852 kW/ (Km^2^)
**Typical temperature difference = 13.9K**
Condenser cost = 7296A0.65, (area in m^2^)
**Re-boiler**
Coefficient of heat transfer = 0.568 kW/ (K m^2^)
**Typical temperature difference = 34.8 K**
**Re-boiler cost = 7296A0.65**
**Vessel Energy cost = energy cost of condenser + energy cost of re-boiler**
**Monitoring price**
**Light pressure steam (6bar, 433K) = $7.78/GJ**
**Medium pressure steam (11bar, 457K) = $8.82/GJ**
**High compressed steam (42bar, 527K) = $9.88/GJ**
**Energy cost of condenser = 0.7172⊆3600⊆8000⊆QC**
**Energy cost of re-boiler = 8000⊆3600⊆monitoring price⊆QR**
**Payback period = 3 years**

### Pressure sensitivity analysis

The data was well presented in S1 Table and S1 Fig, it can be seen that the steady state simulation results are heavily influenced by the thermodynamic model chosen. The study of Liu et al. [31] suggested a pressure extractive distillation strategy as an energy- and cost-efficient method for separating acetone and methanol. This strategy utilized a design based on the non-dominated sorting genetic algorithm. The recommended process from the study resulted in a reduction of CO_2_ emissions and TAC by 48.14% and 22.17%, respectively. Alterations to the thermal conditions of the feed led to different PSD configurations, achieving a 24.08% and 48.33% decrease in CO_2_ emissions compared to traditional extractive distillation technologies. However, extractive distillation methods possess drawbacks, including the introduction of third components that can compromise the final product’s purity [32]. For instance, He et al. [2] research applied butyl butanoate, triethylamine, and butyl propanoate as extractive solvents to separate the methanol-toluene azeotrope. Consequently, the PSD process demonstrates considerable advantages in terms of control and economic feasibility over the extractive method. The premise for the separation task depends on accurate vapor-liquid equilibrium data, which significantly influences the separation of liquid-phase mixtures and the computation of process parameters in chemical production. The foundation for accurately measuring vapour-liquid equilibrium data lies in selecting an appropriate model approach. In this study, the NRTL model was employed for the analysis of the physical property method in a similar system, commonly applied to binary azeotropic systems [33].

The thermodynamic approach to physical properties offers high calculation accuracy, generalization capabilities, and error diagnostic potential. Consequently, employing this calculation model, the study investigates the methanol-toluene azeotrope phase diagram within the pressure range of 0.1–1 MPa. S1 Fig illustrates that the mole fraction of methanol, denoted as Tx, increases from 17% to 3% when the pressure rises from 0.1 MPa to 1 MPa, with a net change of 14%. This variation is considerably greater than that experienced in PSD processes. When the azeotropic composition exhibits a change greater than 5% within the required pressure range, PSD is utilized according to its process separation principle. In the study of Li et al. [34], both partial and full heat integration strategies of PSD were employed to separate methanol and toluene, utilizing chloroform as an entrainer. The process’s feasibility was validated by analyzing residue curve maps. It was observed that the HPC in the stripping section has a steep temperature gradient, which facilitates energy conservation by recycling streams using low-grade heat and by preheating the raw feed. This unique advantage contributed to a 5.39% decrease in the TAC and an 8.32% energy savings in comparison to the extractive distillation process. Furthermore, distillation processes have shown to be effective in separating non-ideal solution systems with relatively good separation effects, indicating that PSD technology can also be applied to such systems.

### Pressure selection

S1 Fig demonstrates the impact of pressure on the azeotropic composition and temperature for the methanol-toluene system across a pressure range of 0.01–10 MPa. In the classical approach to pressure swing rectification, a larger operating pressure differential between the two towers enhances separation, especially at lower pressures where a greater divergence between the vapor and liquid lines suggests a more pronounced azeotropic deviation, which is advantageous for separation [35]. However, with increasing operating pressure differentials, there is a corresponding rise in equipment demands and thus equipment costs. Research on methyl acetate transesterification with iso-butanol as reactive PSD and the binary mixture ethanol/ethyl acetate reveals that the reactive tray nearest to the bottom of the column reaches the highest temperature [11]. By relocating the final reactive stage to upper trays, higher operating temperatures can be achieved. Nevertheless, the azeotropic nature of the mixture, such as the boiling point and the minimum or maximum boiling azeotrope of the output stream, must be considered to ensure the practicability of this adjustment. Conversely, a smaller pressure differential equates to lower equipment costs, yet it complicates separation and escalates the total cost. Therefore, it is crucial to strike a balance between operating and equipment expenses when setting the pressure for the PSD process. Effective rectification requires the low-pressure rectification column’s (LPC’s) azeotropic molar composition to differ by more than 5% from that of the HPC. For the LPC, the principal considerations are the efficiency of the condensing medium in the condenser and maintaining an adequate temperature difference for heat transfer during steam condensation. In contemporary distillation design, pressure parameters are often assumed arbitrarily, despite the significant influence of operating pressure on relative volatilities in azeotropic separation. As a result, operating pressure should be closely monitored due to its critical role in process design and optimization. For instance, Luyben et al. [36] highlighted the importance and the impact of pressure in the PSD process, concluding that energy costs can be substantially reduced by approximately 53% through heat integration and an additional 27% by designing a LPC to operate under vacuum conditions.

The selection of operating pressure has also been underscored by research which optimized process design pressure according to the heating service cost of steam at high, medium, and low pressure [37]. Comparing fully heat-integrated and non-heat-integrated systems, the study showed that full heat integration delivers superior results for separating the minimum-boiling azeotrope ethyl acetate and ethanol, achieving savings of up to 26.64% in TAC, 31.33% in CO_2_ emissions, and 33.33% in energy consumption. However, few studies address the availability of heating services. While discussing pressure optimization outcomes, they often do not elaborate on the rationale behind the achieved results. Generally, it is feasible to conduct separation under normal pressure; for instance, the azeotropic temperature of methanol-toluene is around 63.87°C. Under conditions where cooling water is used for condensation, the primary factor for the HPC is the choice of steam in the reboiler, whereas the LPC must maintain an adequate pressure differential for efficient operation. The Txy phase diagram of methanol-toluene at 0.1 MPa illustrates this point. For the system in question, the operating pressure of the low-pressure tower is 0.1 MPa, with the high-pressure tower exhibiting optimal operating pressures between 0.7 MPa, 0.8 MPa, and 0.9 MPa, as depicted. The operating pressure for the high-pressure tower should therefore be selected from within this prescribed range.

### Process design

#### Conventional pressure swing distillation

Given the characteristics of the methanol-toluene azeotropic system, it is proposed to separate it using two rectification columns operating at different pressures. The process employs two primary columns: a low pressure (LP) and a high-pressure rectification column (HP). A flow diagram is utilized to simulate this rectification system in Aspen Plus. The procedure is as follows: The organic waste liquid, containing methanol and toluene, initially enters the LP column at its midpoint for separation. The LP column’s bottom stream yields a high-purity methanol product, while the top stream produces a low-pressure azeotrope. A portion of this azeotropic stream is recirculated as reflux to the LP column, and another portion is sent to the HP column after being pressurized by a pump. S2 Fig illustrates the double-effect rectification process flow diagram.

## Process flow and simulation regarding the parameters

**Fig 1:**
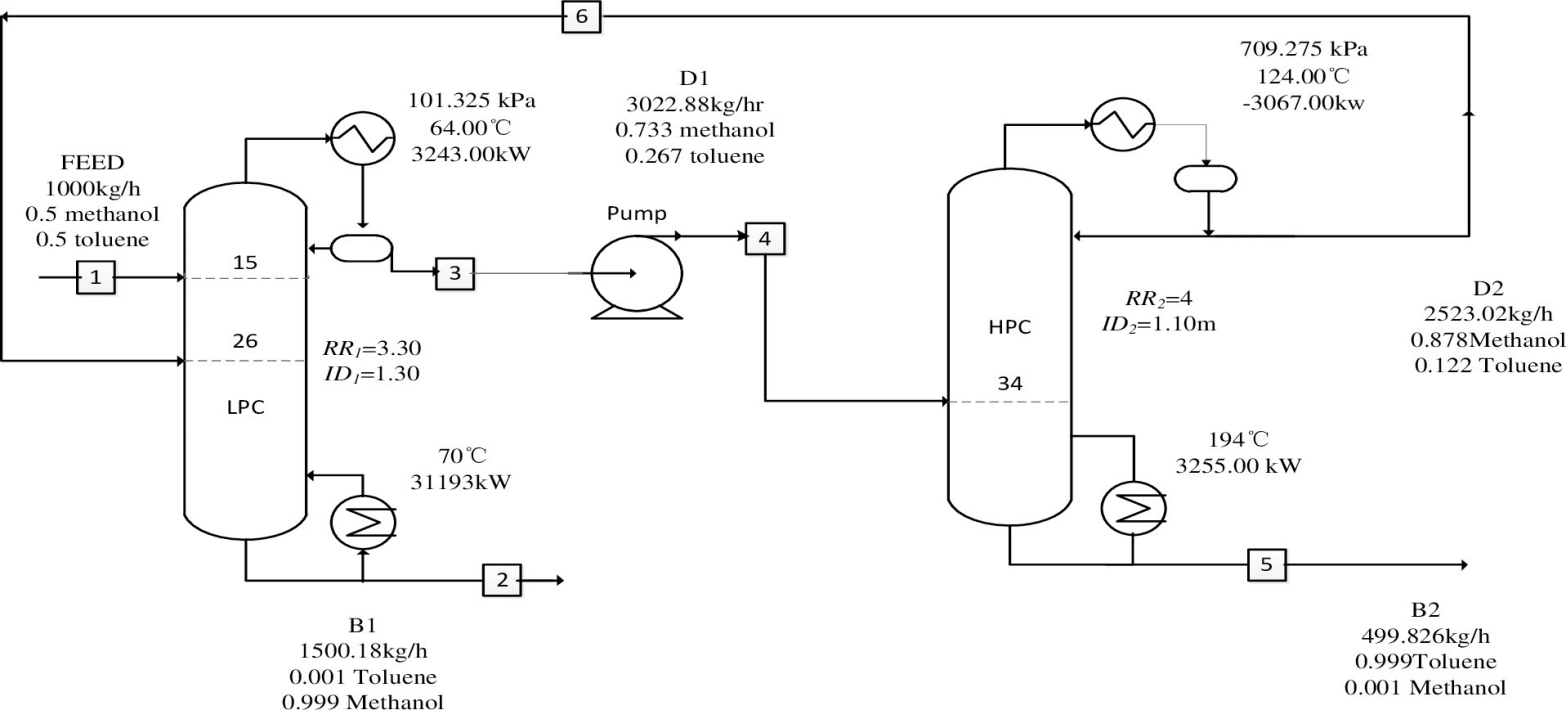
Flow diagram of separating methanol/toluene system by conventional variable-pressure distillation processes.

Compared to other distillation processes such as extractive and azeotropic distillation methods, the PSD process is well-suited for separating close-boiling systems due to its effective selection of entrainers [38]. The work of Iqbal et al. [39] proposed a novel technique for separating pressure-sensitive azeotropes at both maximum and minimum boiling points. The study concluded that the feasible column configuration depends on the type of azeotrope, the pressure effect on the azeotropic material, and the feed composition. It was demonstrated for both systems (minimum and maximum boiling point azeotropes) that sequences of HPC-LPC and LPC-HPC are not universally feasible. Their suitability highly depends on the composition of the feed. The study in examined a three-column PSD process to separate a ternary mixture containing two azeotropes with varying feed compositions [40]. Fig 1 illustrates the process simulation parameters: The feed rate of the methanol/toluene mixture to be separated is 1000 kg/h, with a feeding temperature of 25°C (normal temperature). The mass fractions of methanol and toluene are each 50%. Methanol and toluene products are required to have purities of 99.9% respectively. Given the challenge of constructing energy-efficient and economically viable process designs, the study of Liu et al. [41] proposed and examined reactive PSD for separating a ternary mixture of tetrahydrofuran, methanol, and water.

Considering the composition and characteristics of the methanol-toluene azeotropic system presented above, it is recommended that the system be separated using a combination of two distillation columns operating at different pressures. Mtogo et al. [42] compared the controllability of extractive distillation and the PSD system. The results indicated that the PSD process exhibits significantly better controllability features compared to the other distillation method evaluated. This improved controllability may be attributed to the addition of a third compound as an extractive agent, which increases process complexity. However, while the PSD process requires more energy than extractive distillation, it presents inherent heat integration opportunities between the HPC and the LPC, which could substantially reduce heating energy consumption. Iqbal et al. [39] explored the feasibility of a continuous PSD process and investigated the impact of various feed compositions on column sequencing. Fig 1 displays the flow chart from Aspen Plus simulation software for this system configuration. The system comprises an LPC, a pressure pump, and an HPC. The methanol-toluene feedstock is introduced via stream 1, and the recycle stream is fed into the top of the HPC, as indicated in the diagram. The high-purity methanol product is collected from the bottom stream 3 of the LPC; the LPC top stream product is pressurized by the pump in stream 2, which then feeds into stream 4 of the HPC; and the high-purity methanol product is extracted from stream 5 at the bottom of the HPC. Batches of toluene product are pumped through, with the azeotrope being obtained at low pressure in the LPC overhead stream. Conversely, the overhead stream at high pressure yields an azeotrope.

When it comes to the operating pressures of the two towers, there is a notable impact on the operational costs. The selected operating pressure must comply with the utility’s specifications. Ideally, the temperature difference between the reflux tank and the circulating cooling water for the low-pressure towers should exceed 10°C at the operating pressure. Moreover, Cui et al [43] discussed the optimal selection of operating pressures in various case studies of distillation columns, such as for propylene/propane, benzene/toluene, cyclohexane/cyclohexanol, methanol/water, and the n-pentane/n-hexane/n-heptane system. TAC was estimated for each system using a shortcut method, which was thoroughly presented in the study. Furthermore, Risco et al. [11] examined the selection of pressure for both reactive and non-reactive PSD systems. This paper utilized case studies of methyl acetate transesterification with iso-butanol and the binary mixture of ethanol/ethyl acetate as examples. For the HPC, the temperature of the bottom reboiler should be sufficient to utilize the appropriate grade of steam based on the working pressure. Nevertheless, when defining the pressure for the HPC, the process economics must be taken into account. Generally, the TAC calculation model provided by Luyben et al. [29] is employed to evaluate the process economics. The investigation established that the normal operating pressure for the low-pressure tower is atmospheric and the operational pressure range for the high-pressure tower is 0.61 MPa. The influence of varying the operating pressure of the high-pressure tower on TAC was demonstrated. Energy consumption of the process is minimized when the operating pressure of the high-pressure tower is set to 0.7 MPa, as indicated in S2 Table. Consequently, the operating pressure for the HPC is determined to be 0.7 MPa.

### Assessment of the double-effect distillation process

In the early 1980s in Europe, methanol was considered an energy fuel and was extracted from wood via a destructive distillation process [44]. Subsequently, the distillation method was improved to enable the separation of numerous chemical agents in a cost-effective manner. Multi-effect distillation is one of the most suitable technologies for the distillation process, having been enhanced for large-scale application due to its energy conservation characteristics. According to Putri et al. [45], DED process has demonstrated impressive performance in separating azeotropic mixtures and purifying seawater, offering the advantages of simplicity in operation and significant energy savings. In DED technology, such as in double-effect rectification with two columns, the reboiler of the second column is heated by the vapor from the top of the first column’s reboiler, which can reduce the energy consumption for heating the second column by up to 50%. Thus, in a distillation system employing triple or more columns, the energy savings can be further maximized. For instance, in a triple-effect distillation process with three columns, the heat energy consumption can be reduced by approximately 33%. This is corroborated by the work of Gao et al. [46] which investigated the efficiency of double-effect distillation for separating the N,N-dimethylacetamide and water mixture. The results confirmed that the energy consumption for top mechanical vapor recompression heat pump distillation and the TAC were decreased by up to 32.48% and 30.81%, respectively, while for a DED employing double mechanical vapor recompression heat pumps, energy consumption and TAC were significantly reduced by 78.4% and 47.76%, respectively.

**Fig 2:**
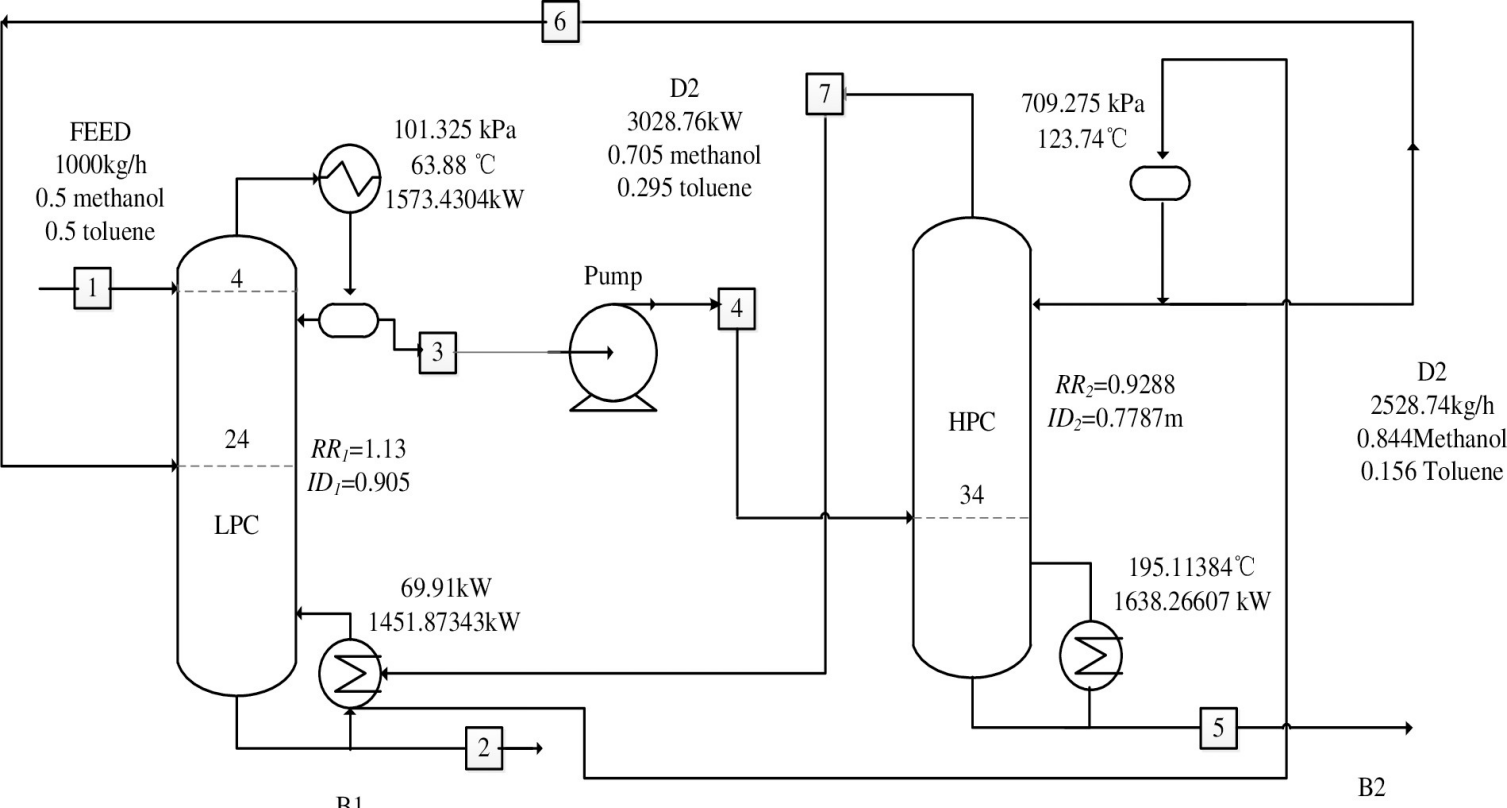
Process flow chart of efficient distillation.

Adjustments in the working pressure between two towers result in a substantial temperature differential, which can be utilized to achieve heat integration. This integration can lower the energy cost of the high-pressure tower’s condenser, consequently reducing overall energy consumption [11]. The subsequent Fig 2 illustrates process flow chart of efficient distillation. As the raw materials are introduced into the LPC via stream 1, the methanol-toluene azeotrope is separated at the column’s top, with the methanol product collected at the bottom; the distilled methanol-toluene azeotropes then proceed to the pressurizing pump through stream 3. After being pressurized to a maximum of 709.725 kPa, the methanol product is obtained at the column’s bottom. The methanol-toluene azeotrope distilled at the top of the tower is routed to stream 6, where the separation continues in a LPC. The temperature of the high-pressure tower overhead condenser, at 123.7°C, is significantly higher than that of the low-pressure tower and kettle reboiler, at 69.9°C. The reboiler heat duty (QR1) at the bottom of the LPC is set equal to the heat duty at the top condenser of the HPC (QC2) by using the "Design Spec" function in the "Flowsheeting Options" in Aspen Plus. RR2 is the manipulated variable, allowing the heat from the condenser at the top of the high-pressure tower to be transferred to the reboiler at the bottom of the low-pressure tower. The calculated TAC of the process is $7.49 × 10^5^ per year.

As depicted in Fig 2, DED is predicated on PSD. The process leverages the latent heat from the condensation of the overhead stream in the HPC to supply heat to the reboiler of the LP, thus diminishing the energy demands of the HPC’s condenser. The system comprises two towers, a low-pressure tower and a high-pressure tower. A significant temperature differential arises between these two towers due to the variance in their operating pressures. This temperature gradient facilitates an integrated energy supply system wherein the condenser at the top of the high-pressure tower is connected to the reboiler at the bottom of the low-pressure tower. By transferring heat to the reboiler of the low-pressure tower, the system effectively reduces energy consumption.

## Results and discussions

Optimization is a systematic process that employs design constraints and criteria, allowing planners to identify the optimal solution. Techniques for optimization have found application across a wide array of fields, addressing a variety of practical challenges. Generally, the goal of optimization is to minimize costs and simultaneously maximize performance, productivity, and efficiency.

### Optimization process

Process optimization is essential for attaining the best optimal parameters and economic feasibility of the new system design. Many studies have been conducted to determine optimization process of distillation technology by using different methods including an algorithm and software package [47], sequential quadratic programming (SQP) [48], stochastic optimization algorithm [49], sequential sensitivity analysis-based technique [50] and harmony search [51]. However, Ma et al. [52] reported sequential sensitivity analysis as inadequate optimal method for complex system. Aspen Plus build on SQP, which allow the congregation of tear streams, inequality and equality constraints simultaneously. Moreover, SQP is logarithmic optimization which uses numerical derivatives for all calculations and tear parameters at every iteration, however it is very challenging to achieve precise gradient information in the system simulation design [53].

**Fig 3:**
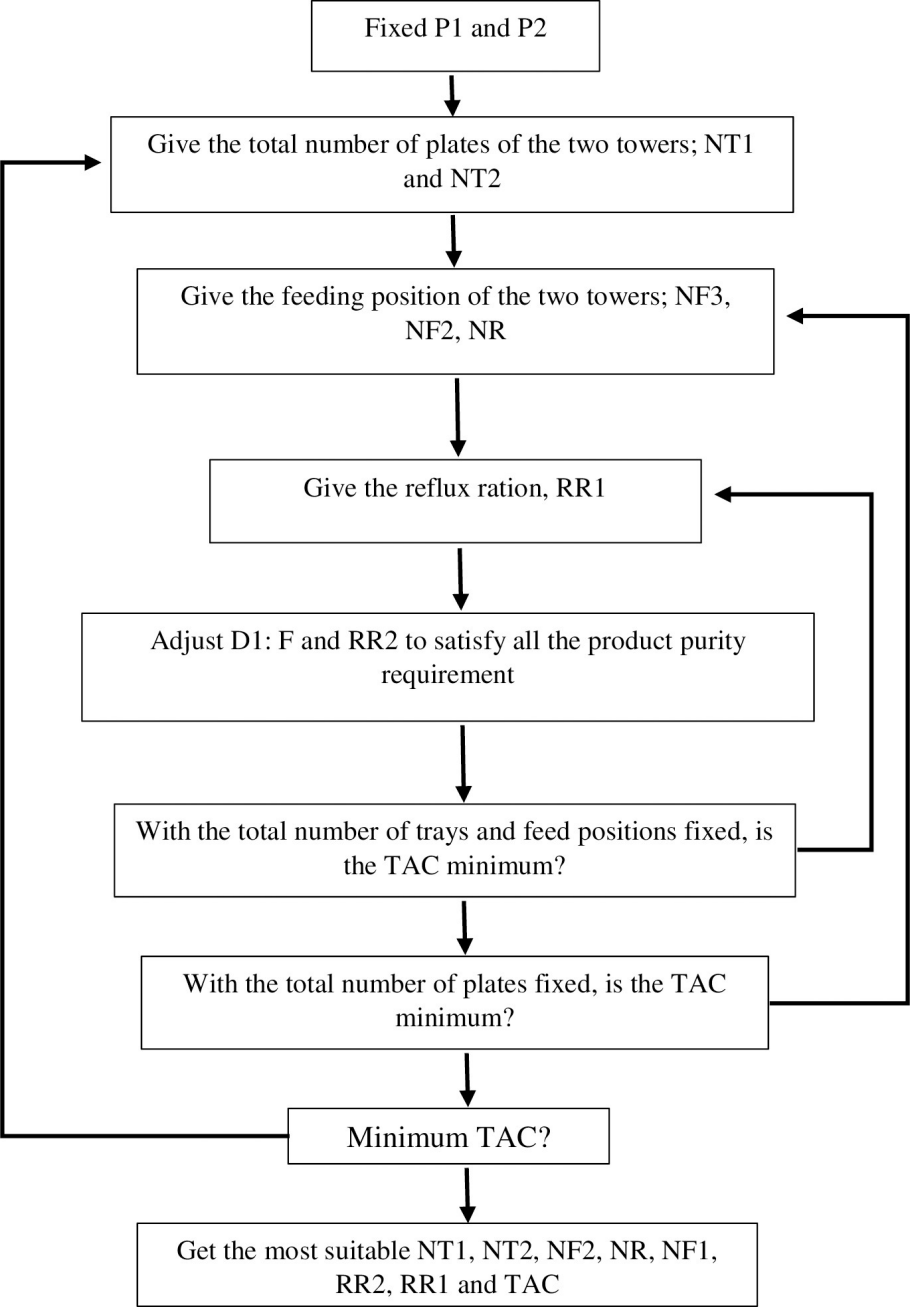
Optimization iterative plan for variable-pressure distillation.

In the PSD process, the optimization iterative method for the separation of methanol and toluene is depicted in Fig 3. During the sequential iterative method’s optimization process, the reflux ratio is identified as having the most significant influence on the TAC, and for this reason, it is selected as the focus of the innermost loop. Subsequently, the feed location is established as the secondary inner cycle, and the total number of trays is designated as the outermost cycle to optimize. The study of Gu et al. [54] examined the optimization of a three-column PSD using a multi-objective genetic algorithm. However, this optimization technique does not guarantee globally optimal solutions. Therefore, it is essential to repeatedly implement stochastic multi-objective genetic algorithms over extended periods, allowing sufficient time for them to converge toward optimal solutions. Zhu et al. [40] used the same method to optimize the triple columns PSD system. The study indicated that, during the optimization process utilizing a genetic algorithm, establishing a link between MATLAB and the simulator via ActiveX technology is essential to prevent interruptions or data loss from computer system or MATLAB crashes. Should such disruptions occur, it is possible to resort to historic data, which must be systematically recorded at each iteration of generation residency time. The optimization sequence involved in the variable PSD process is comprehensively outlined in S3 Table. In the optimization of each manipulated variable layer, the objective function is to determine the optimal value of the manipulated variable. This process uses the minimum TAC as the objective function while maintaining product purity at 99.9%. Among the manipulated variables, there are three related to feeding positions: raw material feeding position, circulating material feeding position, and HPC feeding position. Additionally, there are two variables pertaining to the total number of plates: one for the HPC and one for the LPC.

To achieve the desired product purity in the optimization of methanol/toluene binary system separation in the PSD process, it is vital to utilize the design spec/vary feature in Aspen Plus, which adjusts the controlled variables accordingly. Triwibowo et al. [55] conducted a simulation study using Aspen Plus to purify bioethanol derived from microalgae. The study reported successful attainment of bioethanol purity at 99.9% through the implementation of the PSD process. In a similar vein, the same simulation software was employed to explore the distillation process for trichlorosilane [56]. It has been shown that both full and partial heat integration of the PSD process can decrease the TAC by 27.29% and 15.75%, respectively, when compared to the conventional distillation process. Additionally, Fig 3 illustrates the optimization iterative scheme for variable-pressure distillation. The purity of the methanol product can be increased by altering the top extraction rate (D1/F) of the LPC, and the purity of the benzene product can be increased by modifying the reflux ratio (RR2) of the HPC. The process has six degrees of freedom once the feed conditions and operating pressures of the two columns are determined, including the LPC ’s reflux ratio (RR1), the position where the waste liquid enters the LPC (NF1), and the circulating material enters the LPC (NF2). The position (NR), the position where the material enters the HPC (NF2), the LPC’s total plate number (NT1), and the HPC’s total plate number (NT2) (NT2).

### Influence of the sequence on optimization process

It is required to use the design spec/vary function in Aspen Plus to change the controlled variables in order to obtain the purity of the targeted product while optimizing the separation of the methanol/toluene binary system in the PSA process. The purity of the methanol product reaches 99.9% when the top extraction rate (D1/F) of the LPC is changed; the purity of the benzene product reaches 99.9% when the reflux ratio (RR2) of the HPC is changed. Gu et al. [54] reported the feasibility and separation sequence of multi-objective optimization by the thermodynamic insight via distillation boundaries and the analysis of residue curve. When the feed conditions and operating pressures of the two columns are determined, the process has six degrees of freedom, which include the LPC’s reflux ratio (RR1), the position where the waste liquid enters the LPC (NF1), and the position where the circulating material enters the LPC (NF2). The position (NR), the position where the material enters the HPC (NF2), the LPC’s total plate number (NT1), and the HPC’s total plate number (NT2) (NT2). TAC is commonly used to analyze the economic benefits of the azeotropic system’s separation process system as a significant indication [38].

The recovery rate and reflux ratio of the methanol-toluene azeotrope system are adjusted to keep both methanol and toluene were 99.9% pure, and the sequence block method optimization procedure is carried out to produce the least TAC [57]. The LPC reflux ratio (since the initial process simulation uses the HPC reflux ratio as the manipulated variable, no further optimization is required), the HPC feed position (NF2), the feed position of LPC fresh material (NF1), and the recycle-stream feed position recycle-stream (NR) were used as the inner iterative cycle, and the LPC total trays (NT1) and the HPC total trays (NT2) were used as the outer iterative cycle, according to the iterative sequential optimization method. The operating parameters are optimized, and by optimizing several parameters from the inner loop to the outer loop, the least TAC can be obtained. This research provides a universal PSD optimization method for the methanol-toluene azeotrope system based on this theory, and examines the impact of RR1, NF2, NF1, NR, NT1, and NT2 on TAC.

### Optimization of the pressure swing distillation process

Globally, the most used method to calculate the optimal process design for purity product, energy consumption and economic feasibility is genetic algorithm [58]. The main valuable of this method is selection, mutation and crossover rate. Moreover, as PSD technique regarded as the method of separating binary azeotropes that change composition significantly over a moderate pressure range by using a series of columns operating at different pressures [59], or by adding a separating agent that forms pressure-sensitive azeotrope to separate a pressure-insensitive azeotrope.

### Optimization method and description

The sequential iterative method is employed to improve the process parameters of the methanol-toluene azeotrope system for double-effect rectification in this dissertation, based on the minimum TAC. Afterwards when, the optimal processing parameters are discussed and chosen. The reflux ratio of the LPC (RR1), the reflux ratio of the HPC (RR2), the feed position of the raw material (NF1), the feed position of the HPC (NF2), and the feed of the circulating material are the first seven process parameters to be optimized. Because RR1, RR2 are in the preliminary simulation design regulations (Design specs/Vary) and double-effect rectification design regulations (Flow sheeting Options), NF1, NF2, NR, NT1, NT2 are left to optimize the parameters. The optimization sequence of these five degrees of freedom is addressed using the sequential block iteration technique.

### Optimization of the double effect distillation process

#### Optimization of the reflux

**Fig 4:**
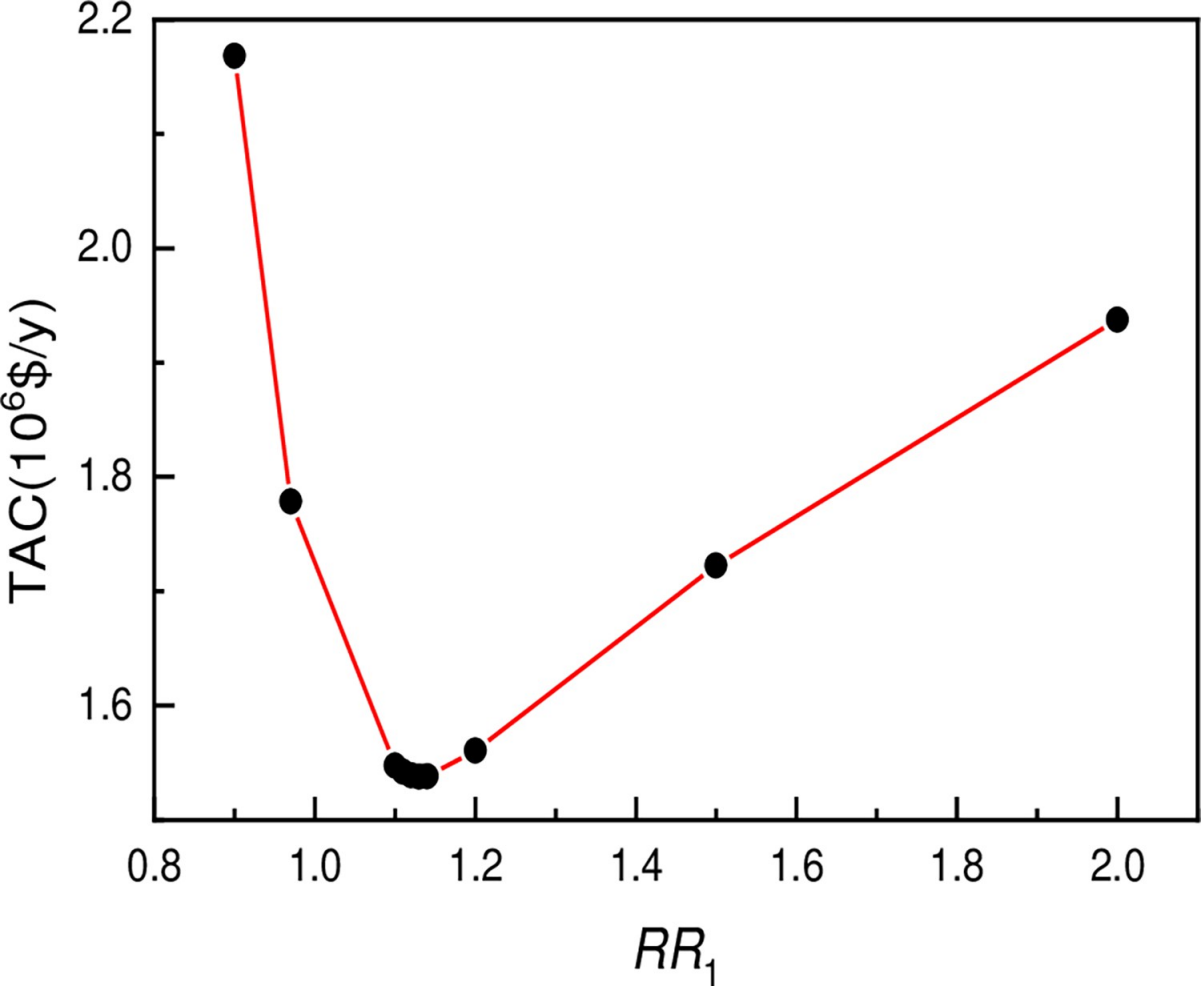
Influence of reflux ratio of low-pressure tower on TAC’.

The PSD optimum parameters are used to obtain separation of azeotropic mixture under the most economic operating conditions. Numerous parameters including pressure, reflux ratio, diameter and temperature can be attuned to optimize TAC [60]. According to studies, the reflux ratio is a significant component in the design and operation of the rectification process, as well as in the separation of PSD processes [61,62]. The influence of reflux ratio of low-pressure tower on TAC is well shown in Fig 4.

**Fig 5:**
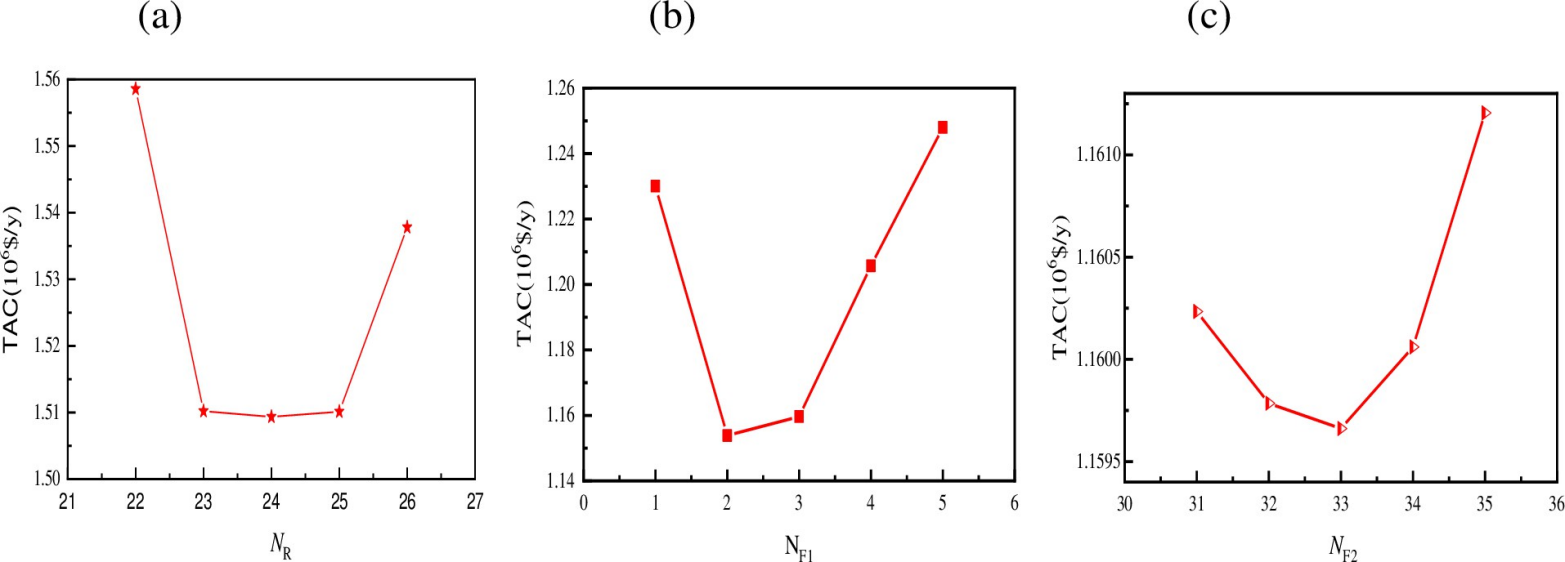
Influence of feeding location on total cost TAC: (a) NF1; (b) NF2; (c) NR.

As a result, the optimization of the reflux ratio takes precedence in this paper. The reflux ratio can be increased properly to make the bottom product purity of the LPC approach the desired result. The smaller the number of theoretical plates and the lower the investment equipment cost, the higher the reflux ratio. However, the higher the reflux ratio, the higher the reboiler running costs and the lower the process design advantage, therefore the reflux ratio must be kept under check [63]. As a result, the reflux ratio is optimized first in the optimization process. The chart shows that as the reflux ratio changes from 0.9 to 2.0, the overall annual cost reduces rapidly at first, then gradually rises. The TAC is the least when the reflux ratio is 1.13. As a result, 1.13 is a good number for the LPC ’s reflux ratio. Fig 5 presents the influence of feeding location on total cost TAC. The influence of optimization sequence of different feeding positions on TAC was well presented in S4 Table, while Fig 5 shows the influence of feeding location on total cost TAC: (a) NF1; (b) NF2; (c) NR.

The main feed position variables for the separation of the methanol and toluene binary azeotrope system in the PSD process include the point where the waste liquid is introduced into the LPC, the position where the circulating material enters the LPC, and the position at the top of the LPC. The material enters the HPC at three different points (NF2). There are a total of six types of optimization sequences based on these three factors, and also S4 Table displays the impacts of optimized sequences of varied feeding positions on the optimum TAC. The TAC achieved from the fifth optimization sequence, that is, first optimize NR, then optimize NF1, and finally optimize NF2, is the smallest, as shown in the table. The sensitivity of the three feed position variables to the influence of the annual total cost TAC can also be seen: NR>NF1>NF2. The impacts of the three feed position variables on the total cost TAC under the optimization sequence are shown in Fig 5A–5C. The TAC lowers initially and then increases as the feeding position changes from top to bottom within a defined range. As a result, the 24th plate is the ideal place for circulating material to reach the low-pressure tower, the third plate is the best place for waste liquid to enter the low-pressure tower, and the 33rd plate is the optimum place for material to enter the high-pressure tower.

#### Optimization of the total number of trays

Energy consumption and capital material investment are significantly impacted by the number of trays utilized in the optimization process. Therefore, a judicious selection of the number of trays is essential to minimize the TAC. Various researchers have developed optimization techniques for both non-equilibrium and equilibrium reactive distillation columns by employing mathematical programming models [51,52]. The pioneers of the tray-by-tray mathematical model are referenced in [64]. Their mixed integer nonlinear programming model was solved using the generalized Benders decomposition algorithm as detailed in [65]. The optimization of the total number of trays is achieved by multiplying a binary variable, which indicates the presence of a tray, with related constraints. This introduces bilinear relationships that complicate the process, resulting in poor numerical performance and resolution challenges. A stable and efficient decomposition system for solving optimization problems involving differential algebraic equations was well-represented in the literature [66].

Orthogonal collocation is used within a sparse rSQP framework in order to obtain the control profiles and the parameters given a fixed element placement. They have approved that, compared to the previous approaches, the sparse decomposition of the discretized system is more efficient. In addition, this method allows detection of unstable modes by simple selection of pivots. Only a few studies have been conducted in the dynamic optimization area. Cervantes et al. [66] have solved well-known unstable challenges, including a plug-flow reactor model and dynamic index one optimization problems for both batch and continuous reactive distillation columns. The total number of trays has a significant impact on the distillation column’s separation efficiency. When the same separation effect is achieved, the outer layer is utilized to iteratively circulate the number of trays until the optimal number of trays is found [67]. The effect of the theoretical plate number of the high- and LPC’s on the TAC of the methanol-toluene PSD separation system was explored separately while keeping other simulation conditions constant. Souza et al. [68] presented the optimization of the design of distillation column trays. The total plate number of the two columns was selected as the outermost loop in the optimization iteration procedure to accomplish global optimization. The influence of the optimal sequence of different plate numbers on TAC are listed in S5 Table. The optimal sequence, as shown in the table, the fifth optimization sequence: that is, the theoretical plate number NT1 of the LPC is optimized first, followed by the theoretical plate number NT2 of the HPC, and the obtained TAC is 1.159453×10^6^$/y, where TAC is the smallest among the other sequences. It can be shown that the theoretical plate number of the two towers is more sensitive to TAC: NT1>NT2. The variation trend of TAC with the number of theoretical plates during the fifth optimization process is shown in the table. Additionally, as the number of trays increases, the TAC first decreases and then increases.

#### Results and energy saving analysis of optimization

The comparison of the economic performance before and after optimization of the conventional PSD can be seen in S6 Table. The TAC of the traditional PSD process has been lowered by 49.50% after optimization compared to before optimization, and the energy cost has decreased by 53.50%. According to the optimization results, the temperature difference between the top stream of the high-pressure tower and the bottom stream of the low-pressure tower is 54 degrees, and the corresponding heat loads are -1305.00 kW and 1479.00 kW, respectively. These two cold and hot streams can be used for heat exchange to achieve heat integration and save energy.

When compared to the conventional PSD process, the TAC of some heat-integrated PSD processes decreased by 33.10%, the equipment cost decreased by 18.80%, and the energy consumption cost decreased by 39.10%, resulting in significant energy and cost savings.

#### Optimization sequence of double effect distillation

The raw feed containing the mixed form of methanol and toluene undergoes pre-treatment which includes removal or reduction of any impurities that may interfere with the efficiency or safety standards. In this stage, operated normally under high pressure condition using first distillate column, separates majority fraction based on their volatility differences at given pressures and temperature levels resulting into top product rich in one component. The product goes through de-pressuring step where it’s exposed to lower different-pressure level from previous HPC causing immediate evaporation due reducing boiling points as per swing technique; driving secondary parting action enhancing purity level further. LPC unpressured vapor streams now enter second column operating typically at low pressures compared initial conditions for yet another round precise fractional separation obtaining bottom pure discharge stream ideally stripped off remaining minority element present inside blend mixtures after exiting HPC tower initially kickstarting more complete isolations effectively.

**Fig 6:**
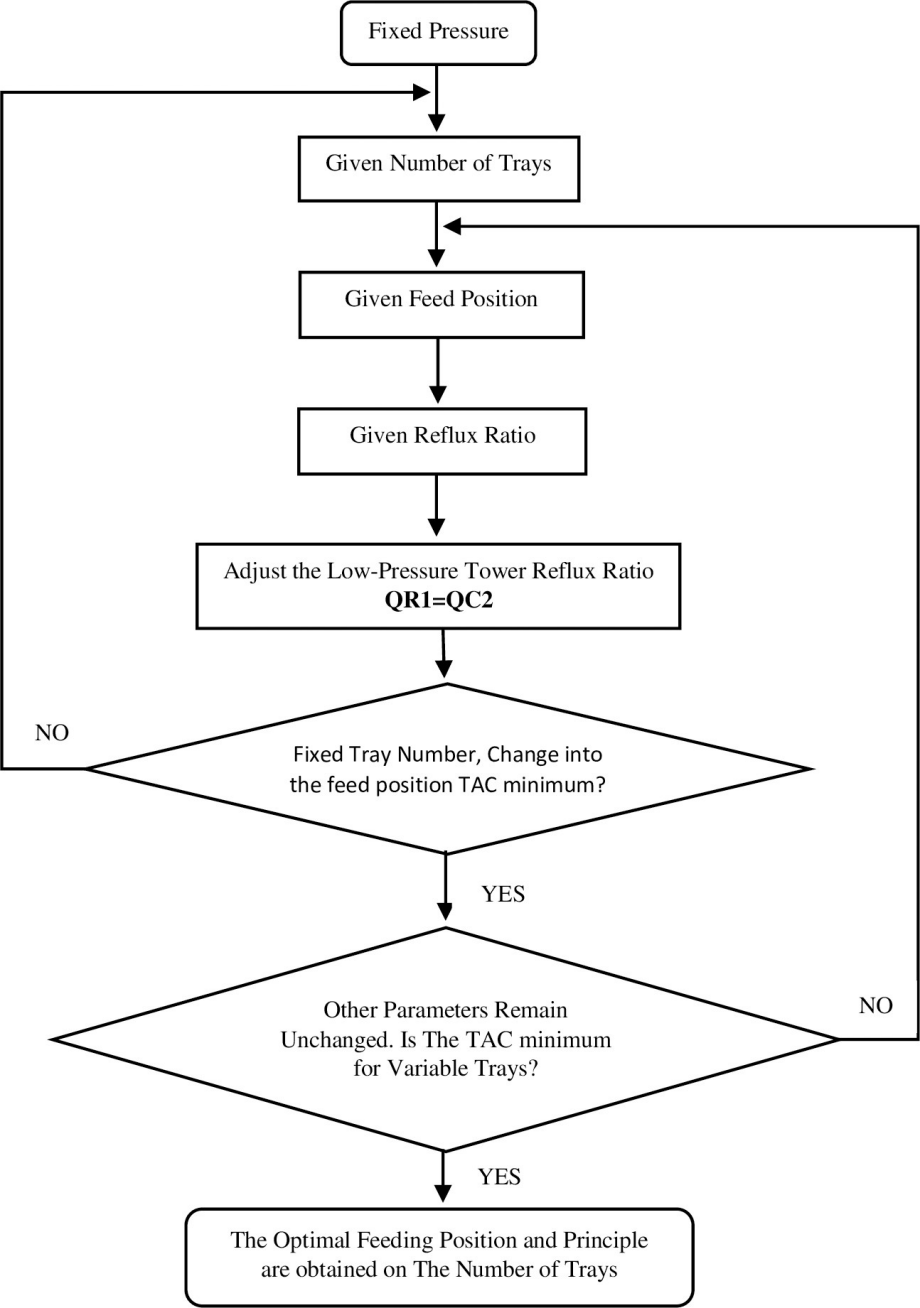
Optimization model of double-effect distillation.

Overhead condenser attached above LP towers recycles back liquid phase constituents not required directly into higher pressurized primary system reprocessing them again improving overall yields while minimizing unavoidable wastage scenario commonly connected within most chemical engineering operations addressing sustainability concerns indirectly too beside just merely performing its standard cyclic unit tasks specifically related compound segregating activities only. High-purity separated liquids collected respectively from corresponding columns stored safely while ensuring optimal duty cycles maintained adjusting key parameters digitally sometimes to accommodate variable input feedstock compositions and fluctuations balancing system integrity plus profitability equilibrium together. In addition, any gases, solids or liquids that are residual from separation process should be treated appropriately before release into the environment in accordance with environmental standards and regulations. The optimization model of double-effect distillation was well presented in Fig 6.

The outer iterative loops NT1, NT2 and the inner iterative loops NF1, NF2, NR are used in the optimization process. Select seven different optimized sequences for optimization (see optimized sequences on S7 Table for optimization sequence of DED) to be able to find the optimal process parameters in this paper, supposing that the feed position is optimized first and then the number of trays is optimized (see the optimization block diagram in Fig 6 on the optimization model of DED). W. Chen et al. [69] method was based on the NSGA- III algorithm, which is implemented through a hybrid platform using Python v3.9 and Aspen Plus v11for optimization of the methanol distillation process. When a complete heat integration is designed using PSD, the latent heat of condensation from the HPC’s top stream is used to heat and provide energy to the LPC’s reboiler, saving the HPC’s condenser’s energy cost. The optimization approach is also different in the whole thermal integration process, which employs the sequential block method optimization to determine the best optimization sequence and process parameters. The LPC’s theoretical plate number (NT1) is the outer iterative cycle, while the raw material feed position (NF1), the circulating stream feed position (NR), the feed position of the HPC (NF2), and the HPC’s reflux ratio (RR2) are the inner layers iterative loop. The heat source energy supply system for the latent heat of vaporization of the (LP) reboiler is the latent heat of vapor condensation at the top of the (HP) tower. The reboiler is regarded as a unit.

### Optimization of the pressure swing distillation parameters

#### Influence of the feeding position

**Fig 7:**
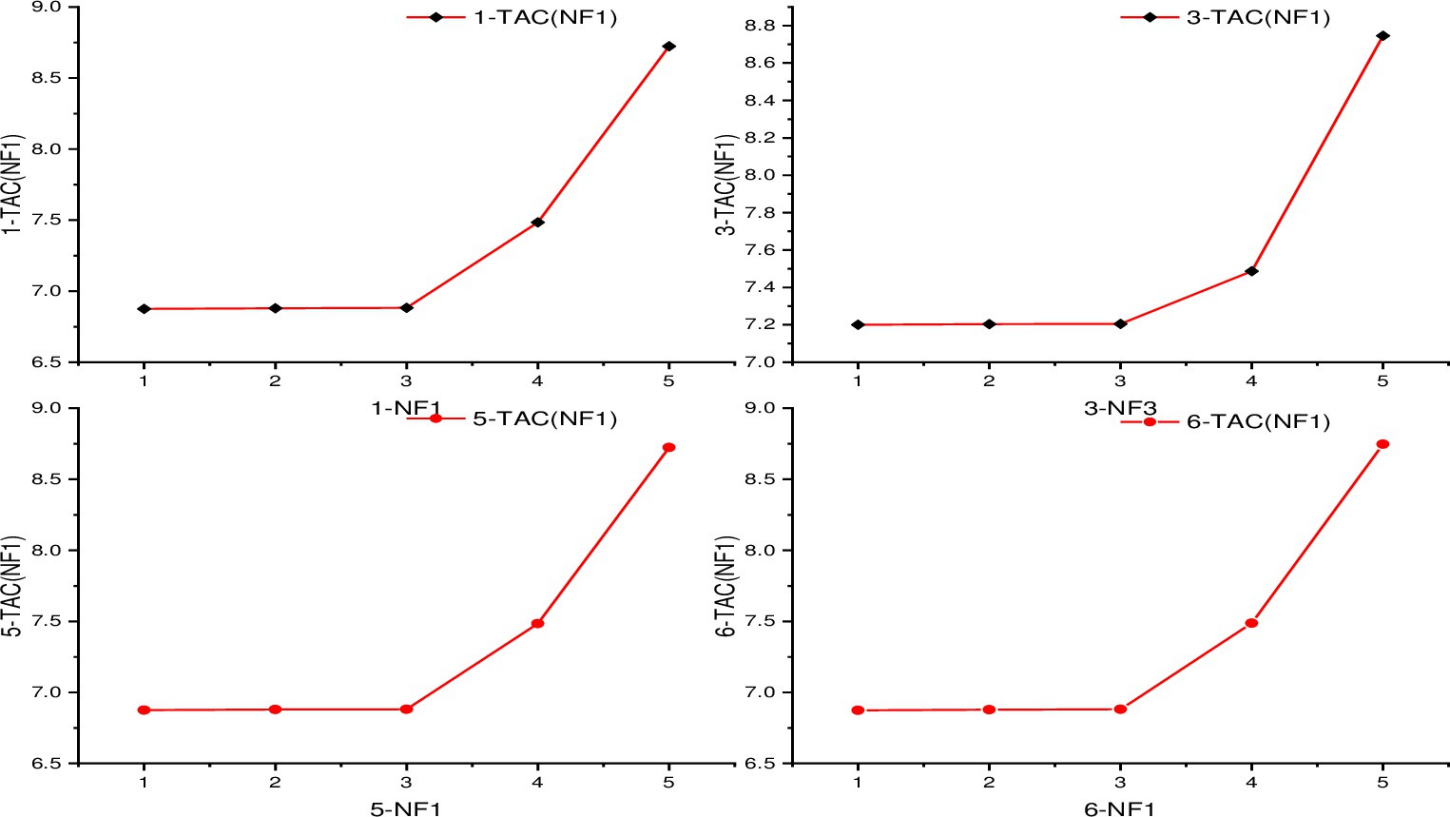
Effect of feed location of different sequences on TAC.

The sequence inertial module approach is utilized as the optimization method to optimize the feeding position since it has a significant impact on productivity while keeping other process parameters constant and TAC is affected by the varied feeding positions used in the optimization procedure [67]. The content of non-volatile components at the top of the tower will be high if the feeding position is too high, while the content of volatile components at the bottom of the column will grow if the feeding position is too low. As a result, selecting a proper feeding posture is critical to the entire feeding process. Fig 7 shows the effect of feed location of different sequences on TAC and S8 Table presents data charts for the figures. As can be observed from the figure, the TAC of the seven sequences is steady at first and then increases dramatically as the feed plate moves downward. The 1st, 2nd, and 3rd trays have a huge TAC. The first theoretical tray cannot be fed at this position since it is a condenser and the second tray lacks safety and stability. As a result, the third tray is chosen. A tray was used as the optimal feed location for the feeding position of all seven sequences.

#### The number of the plates

**Fig 8:**
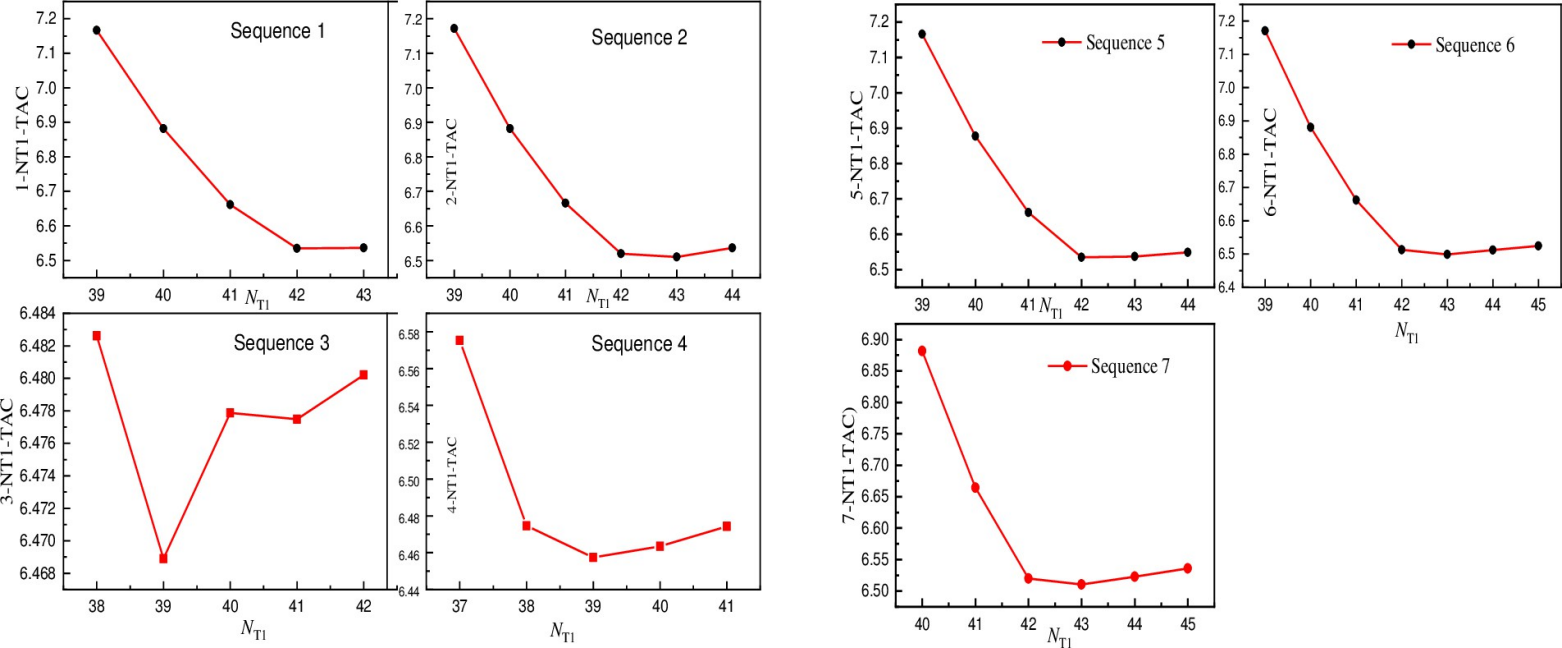
Influence of NT1 on TAC.

The number of plates or their numbering is a critical parameter in the simulation process [70]. The impact of NT1 on TAC is depicted in Fig 8. The sequence 1, 2, 4, 5, 6, 7 gradually reduces with the number of theoretical plates, as indicated in the picture, and then tends to be stable. The optimal number of theoretical plates is 42, 43, 39, 42, 43, 43; the TAC of sequence 3 decreased at first, then grew abruptly, and eventually increased progressively as the number of plates increased, with 39 being the optimal number of plates.

As the number of plate’s increases, the effect of N_T2_ TAC gradually declines and then increases and the optimal plate numbers of the series are 39, 41, 41, 39, 39, 39, 40, and 40. To summarize, the optimal sequence is 4 and the optimized values are provided in S9 Table, TAC can be saved by 3.8% if a comparison is made between before and after optimization.

#### Environmental evaluation

From an environmental perspective, PSD is more conducive to reduce carbon footprint. This study found that, the HPC in the stripping section has a steep temperature gradient, which benefits energy savings by using low-grade heat recovery streams and preheating the feedstocks. This unique property contributes to 8.32% energy savings and also after process optimization (see S6 Table) the energy cost has decreased by 53.50%. Hence, the PSD requires less energy compared to traditional distillation methods which require high heat input that contributes significantly to global warming. Mariem et al. [71] studied an environmental factors of heat pump-assisted PSD of maximum-boiling azeotropic mixture water-ethylenediamine. The results show that, the heat integration decreased the CO_2_ emissions by 19.5% using partial heat integration and by 20.6% using full heat integration. Moreover, PSD reduce waste into eco-system, as it does not require the introduction of third component during the distillation process. And also, the optimization of this process leads to highly efficient in separating complex mixtures than other techniques, it decreases waste production significantly; thus, reducing pollution levels.

Furthermore, compared with traditional processes such as fractional distillation or liquid-liquid extraction, the emission of carbon resulting from PSD tends to be lower due its reduced power requirements and elimination of need for additional solvents or materials involved in these processes, hence contributing less greenhouse gases emission. Luo et al. [72] compared extractive distillation and fully heat-integrated PSD with 2- methoxyethanol as an entrainer. In addition, this study also investigated the dynamic control and optimal design of the two processes respectively. It was reported that, the fully heat-integrated PSD system offers 5.75% reduction in the TAC and 7.97% saving in energy consumption as compared to the extractive distillation system. Additionally, PSD achieve higher recovery rates through utilization and optimization of this technology (as 99.99% of azeotrope mixture can be recovered) when compared against alternative approaches requiring costly chemicals/solvent use along with their appropriate subsequent disposal measures being taken care of effectively, offers another positive implication on environment conservation.

## Conclusion

In this study, we optimized various parameters to assess their influence on an azeotropic system during the PSD process, using the methanol/toluene system as a case study. This optimization was conducted using the Aspen Plus simulation software and Origin, with a particular focus on TAC for calculations and diagrams. This technique proved effective for separating the methanol/toluene binary azeotrope and yielding purer products. The azeotrope formed by methanol and toluene was analyzed for pressure sensitivity, with the low-pressure tower operational pressure set at 101.325 kPa and the high-pressure tower operating pressure set at 709.275 kPa. Simulation results indicate that employing high-pressure and low-pressure columns for azeotrope separation can be economical, owing to the energy-saving benefits of double-effect rectification. The product obtained from the bottom of the low-pressure tower is methanol, with a purity approaching 99.9%, while the product from the high-pressure tower kettle is toluene, which can also achieve a purity of approximately 99.9%. Following the optimization of the conventional pressure swing rectification procedure, the heat exchange between the top stream of the high-pressure tower and the bottom stream of the low-pressure tower facilitates heat integration, thereby reducing energy consumption. Calculations show that the PSD process, after partial heat integration, can decrease the TAC by 33%, equipment costs by 19%, and energy consumption costs by 39% compared to the optimized conventional PSD process, resulting in significant energy savings and economic benefits. In comparing the TAC of conventional pressure swing rectification with double-effect rectification, it is essential to heat the reboiler of the low-pressure tower kettle using the condensing heat from the top of the high-pressure tower. This approach achieves a 65% cost reduction, serving as a benchmark for the design and optimization of separation processes in PSD.

## Supporting information

S1 Fig Influence of pressure on azeotropic composition and temperature of methanol/toluene system vs T/MPa. (DOCX)

S2 Fig Double-effect rectification process flow. (DOCX)

S1 Table The calculation of the T/MPA vs Temperature and Toluene Molar. (DOCX)

S2 Table Economic comparison of different operating pressures of high-pressure columns. (DOCX)

S3 Table Optimization sequences involved in the variable pressure distillation process. (DOCX)

S4 Table Influence of optimization sequence of different feeding positions on TAC. (DOCX)

S5 Table Influence of optimal sequence of different plate number on TAC. (DOCX)

S6 Table Comparison of economy of conventional variable-pressure distillation before and after optimization and partial thermal integrated variable-pressure distillation. (DOCX)

S7 Table Optimization sequence of double-effect distillation. (DOCX)

S8 Table Data chart for the diagram. (DOCX)

S9 Table Comparison of parameters before and after optimization. (DOCX)
