# Supplementary material for: A simulation study on the process design and optimization pressure swing separation of azeotropic mixture methanol and toluene
Source: PLoS One. 2024 Dec 23;19(12):e0310541. doi: 10.1371/journal.pone.0310541 (PMC11666024; doi:10.1371/journal.pone.0310541)
Supplement: S1 Fig — (DOCX) [file pone.0310541.s001.docx]

**Figure S1. Influence of pressure on azeotropic composition and temperature of methanol/toluene system vs T/MPa**
