# Supplementary material for: A simulation study on the process design and optimization pressure swing separation of azeotropic mixture methanol and toluene
Source: PLoS One. 2024 Dec 23;19(12):e0310541. doi: 10.1371/journal.pone.0310541 (PMC11666024; doi:10.1371/journal.pone.0310541)
Supplement: S1 Table — (DOCX) [file pone.0310541.s003.docx]

**Table S1: The calculation of the T/MPA vs Temperature and Toluene Molar**

| **T/MPa (A)** | **Temperature (B)** | **Toluene Molar (D)** |
| --- | --- | --- |
| 0.01 | 14.54 | 0.1662 |
| 0.1 | 63.87 | 0.113 |
| 0.2 | 82.79 | 0.0915 |
| 0.3 | 95.01 | 0.0772 |
| 0.4 | 104.24 | 0.0662 |
| 0.5 | 111.76 | 0.0571 |
| 0.6 | 118.15 | 0.0494 |
| 0.7 | 123.72 | 0.0424 |
| 0.8 | 128.69 | 0.0362 |
| 0.9 | 133.18 | 0.0306 |
| 1 | 137.28 | 0.0254 |
