# Supplementary material for: A simulation study on the process design and optimization pressure swing separation of azeotropic mixture methanol and toluene
Source: PLoS One. 2024 Dec 23;19(12):e0310541. doi: 10.1371/journal.pone.0310541 (PMC11666024; doi:10.1371/journal.pone.0310541)
Supplement: S2 Table — (DOCX) [file pone.0310541.s004.docx]

**Table S2: Economic comparison of different operating pressures of high-pressure columns**

| **Operating parameters** | **Preliminary process simulation of pressure swing distillation in Methanol-Toluene System** | | | | |
| --- | --- | --- | --- | --- | --- |
| P_1_/P_2_ (MPa) | 0.1/0.6 | 0.1/0.7 | 0.1/0.8 | 0.1/0.9 | 0.1/1 |
| RR_1_ | 3.7 | 3.3 | 5.51 | 5.45 | 4.52 |
| RR_2_ | 2.77 | 4 | 5.1 | 5 | 5.2 |
| N_T1_ | 36 | 40 | 40 | 42 | 44 |
| N_T2_ | 35 | 40 | 40 | 42 | 41 |
| N_F1_ | 19 | 15 | 21 | 29 | 16 |
| N_F2_ | 13 | 32 | 23 | 20 | 19 |
| N_R_ | 23 | 25 | 20 | 19 | 20 |
| D_1_/m | 1.58 | 1.30 | 1.54 | 1.52 | 1.40 |
| D_2_/m | 1.22 | 1.10 | 1.31 | 1.09 | 1.22 |
| Recovery rate D/F1 | 0.8715 | 0.8334 | 0.8211 | 0.8193 | 0.818 |
| Recovery rate D/F2 | 0.9486 | 0.9302 | 0.92392 | 0.923052 | 0.92252 |
| TAC (×$106/y) | 2.8098 | 2.2967 | 3.2003 | 2.7057 | 2.5379 |
