# Supplementary material for: A simulation study on the process design and optimization pressure swing separation of azeotropic mixture methanol and toluene
Source: PLoS One. 2024 Dec 23;19(12):e0310541. doi: 10.1371/journal.pone.0310541 (PMC11666024; doi:10.1371/journal.pone.0310541)
Supplement: S3 Table — (DOCX) [file pone.0310541.s005.docx]

**Table S3: Optimization sequences involved in the variable pressure distillation process**

| **Serial number** | **Step 1** | **Step 2** | **Step 3** | **Step 4** | **Step 5** | **Step 6** |
| --- | --- | --- | --- | --- | --- | --- |
| **1** | RR_1_ | N_F1_ | N_F2_ | N_R_ | N_T1_ | N_T2_ |
| **2** | RR_1_ | N_F1_ | N_R_ | N_F2_ | N_T1_ | N_T2_ |
| **3** | RR_1_ | N_F2_ | N_F1_ | N_R_ | N_T1_ | N_T2_ |
| **4** | RR_1_ | N_F2_ | N_R_ | N_F1_ | N_T1_ | N_T2_ |
| **5** | RR_1_ | N_R_ | N_F1_ | N_F2_ | N_T1_ | N_T2_ |
| **6** | RR_1_ | N_R_ | N_F2_ | N_F1_ | N_T1_ | N_T2_ |
| **7** | RR_1_ | N_F1_ | N_F2_ | N_R_ | N_T2_ | N_T1_ |
| **8** | RR_1_ | N_F1_ | N_R_ | N_F2_ | N_T2_ | N_T1_ |
| **9** | RR_1_ | N_F2_ | N_F1_ | N_R_ | N_T2_ | N_T1_ |
| **10** | RR_1_ | N_F2_ | N_R_ | N_F1_ | N_T2_ | N_T1_ |
| **11** | RR_1_ | N_R_ | N_F1_ | N_F2_ | N_T2_ | N_T1_ |
| **12** | RR1 | N_R_ | N_F2_ | N_F1_ | N_T2_ | N_T1_ |
