# Supplementary material for: A simulation study on the process design and optimization pressure swing separation of azeotropic mixture methanol and toluene
Source: PLoS One. 2024 Dec 23;19(12):e0310541. doi: 10.1371/journal.pone.0310541 (PMC11666024; doi:10.1371/journal.pone.0310541)
Supplement: S4 Table — (DOCX) [file pone.0310541.s006.docx]

**Table S4: Influence of optimization sequence of different feeding positions on TAC**

| **Minimum TAC for different sequences** | **Optimization variables** | | |
| --- | --- | --- | --- |
|  | ***N*_F1_** | ***N*_F2_** | ***N*_R_** |
| TAC1 (×106$/y) | 1.4676 | 1.529612 | 1.284557 |
| TAC2 (×106$/y) | 1.4736 | 1.284064 | 1.285641 |
| TAC3 (×106$/y) | 1.4676 | 1.529612 | 1.284557 |
| TAC4 (×106$/y) | 1.1554 | 1.539775 | 1.502307 |
| TAC5 (×106$/y) | 1.1596 | 1.159660 | 1.509349 |
| TAC6 (×106$/y) | 1.1554 | 1.502277 | 1.509349 |
