# Supplementary material for: A simulation study on the process design and optimization pressure swing separation of azeotropic mixture methanol and toluene
Source: PLoS One. 2024 Dec 23;19(12):e0310541. doi: 10.1371/journal.pone.0310541 (PMC11666024; doi:10.1371/journal.pone.0310541)
Supplement: S5 Table — (DOCX) [file pone.0310541.s007.docx]

**Table S5: Influence of optimal sequence of different plate number on TAC**

| **1** | **38** | 1.290164 | **41** | 1.281026 | **38** |
| --- | --- | --- | --- | --- | --- |
| **2** | **39** | 1.282509 | **41** | 1.282380 | **39** |
| **3** | **38** | 1.290164 | **41** | 1.281026 | **38** |
| **4** | **40** | 1.161188 | **41** | 1.160630 | **40** |
| **5** | **41** | 1.159619 | **39** | 1.159453 | **41** |
| **6** | **40** | 1.161173 | **41** | 1.160630 | **40** |
| **7** | **38** | 1.281026 | **41** | 1.283631 | **38** |
| **8** | **38** | 1.281411 | **41** | 1.284012 | **38** |
| **9** | **38** | 1.281055 | **41** | 1.283633 | **38** |
| **10** | **40** | 1.160630 | **41** | 1.160630 | **40** |
| **11** | **41** | 1.159679 | **39** | 1.159843 | **41** |
| **12** | **40** | 1.160630 | **41** | 1.160630 | **40** |
