# Supplementary material for: A simulation study on the process design and optimization pressure swing separation of azeotropic mixture methanol and toluene
Source: PLoS One. 2024 Dec 23;19(12):e0310541. doi: 10.1371/journal.pone.0310541 (PMC11666024; doi:10.1371/journal.pone.0310541)
Supplement: S6 Table — (DOCX) [file pone.0310541.s008.docx]

**Table S6: Comparison of economy of conventional variable-pressure distillation before and after optimization and partial thermal integrated variable-pressure distillation**

| Parameter | Traditional pressure swing distillation process (before optimization) | | Traditional pressure swing distillation process (after optimization) | | Partially heat-integrated pressure swing distillation process | |  |
| --- | --- | --- | --- | --- | --- | --- | --- |
|  | **High-pressure tower** | **Low-pressure tower** | **High-pressure tower** | **Low-pressure tower** | **High-pressure tower** | **Low-pressure tower** |  |
| Total number of trays | 40 | 40 | 39 | 41 | 39 | 41 | |
| Feeding position | 32 | 15/26 | 33 | 3/33 | 33 | 3/33 | |
| Reflux ratio | 4 | 3.3 | 4 | 1.13 | 4 | 1.13 | |
| Condenser temperature (°C)/  Heat load (kW) | 124/  -3067 | 64/  -3243 | 124/  -1305 | 64/  -1604 | 124/  -1305 | 64/  -1604 | |
| Reboiler temperature (°C)/  Heat load (kW) | 194/  3255 | 70/  3119 | 195/  1495 | 70/  1479 | 195/  1495 | 70/  1479 | |
| Total cost (106$/y) | 2.2967 | | 1.1595 | | 0.775703 | | |
| Energy cost (106$/y) | 1.7554 | | 0.81689 | | 0.497469 | | |
| Equipment cost (106$/y) | 1.6240 | | 1.0278 | | 0.834701 | | |
