# Supplementary material for: A simulation study on the process design and optimization pressure swing separation of azeotropic mixture methanol and toluene
Source: PLoS One. 2024 Dec 23;19(12):e0310541. doi: 10.1371/journal.pone.0310541 (PMC11666024; doi:10.1371/journal.pone.0310541)
Supplement: S7 Table — (DOCX) [file pone.0310541.s009.docx]

**Table S7:** **Optimization sequence of double-effect distillation**

| **Serial number** | **Sequence** |
| --- | --- |
| 1 | $\text{N}_{\text{F2}}\text{→}\text{N}_{\text{R}}\text{→}\text{N}_{\text{F1}}\text{→}\text{N}_{\text{T2}}\text{→}\text{N}_{\text{T1}}$ |
| 2 | $\text{N}_{\text{F2}}\text{→}\text{N}_{\text{R}}\text{→}\text{N}_{\text{F1}}\text{→}\text{N}_{\text{T1}}\text{→}\text{N}_{\text{T2}}$ |
| 3 | $\text{N}_{\text{F2}}\text{→}\text{N}_{\text{F1}}\text{→}\text{N}_{\text{R}}\text{→}\text{N}_{\text{T2}}\text{→}\text{N}_{\text{T1}}$ |
| 4 | $\text{N}_{\text{F1}}\text{→}\text{N}_{\text{R}}\text{→}\text{N}_{\text{F2}}\text{→}\text{N}_{\text{T1}}\text{→}\text{N}_{\text{T2}}$ |
| 5 | $\text{N}_{\text{R}}\text{→}\text{N}_{\text{F2}}\text{→}\text{N}_{\text{F1}}\text{→}\text{N}_{\text{T2}}\text{→}\text{N}_{\text{T1}}$ |
| 6 | $\text{N}_{\text{R}}\text{→}\text{N}_{\text{F1}}\text{→}\text{N}_{\text{F2}}\text{→}\text{N}_{\text{T1}}\text{→}\text{N}_{\text{T2}}$ |
| 7 | $\text{N}_{\text{R}}\text{→}\text{N}_{\text{F2}}\text{→}\text{N}_{\text{F1}}\text{→}\text{N}_{\text{T1}}\text{→}\text{N}_{\text{T2}}$ |
