# Supplementary material for: A simulation study on the process design and optimization pressure swing separation of azeotropic mixture methanol and toluene
Source: PLoS One. 2024 Dec 23;19(12):e0310541. doi: 10.1371/journal.pone.0310541 (PMC11666024; doi:10.1371/journal.pone.0310541)
Supplement: S8 Table — (DOCX) [file pone.0310541.s010.docx]

**Table S8: Data chart for the diagram**

| **1-NF1** | **1-TAC(NF1)** | **3-NF3** | **3-TAC(NF1)** | **5-NF1** | **5-TAC(NF1)** | **6-NF1** | **6-TAC(NF1)** |
| --- | --- | --- | --- | --- | --- | --- | --- |
| 1 | 6.87588 | 1 | 7.20083 | 1 | 6.87581 | 1 | 6.87409 |
| 2 | 6.87929 | 2 | 7.20349 | 2 | 6.87923 | 2 | 6.87806 |
| 3 | 6.88191 | 3 | 7.20531 | 3 | 6.88174 | 3 | 6.88078 |
| 4 | 7.48449 | 4 | 7.48656 | 4 | 7.48376 | 4 | 7.48676 |
| 5 | 8.72292 | 5 | 8.74585 | 5 | 8.72283 | 5 | 8.7467 |
