# Supplementary material for: A simulation study on the process design and optimization pressure swing separation of azeotropic mixture methanol and toluene
Source: PLoS One. 2024 Dec 23;19(12):e0310541. doi: 10.1371/journal.pone.0310541 (PMC11666024; doi:10.1371/journal.pone.0310541)
Supplement: S9 Table — (DOCX) [file pone.0310541.s011.docx]

| **Parameter** | **Double effect distillation** | **After double-effect rectification optimization** |
| --- | --- | --- |
| *RR*_1_ | 1.131 | 0.7885 |
| *N*_F1_/NR | 4/24 | 3/19 |
| *N*_T1_ | 40 | 39 |
| *ID*_1_(m) | 0.905 | 0.83 |
| *QC*_1_(kW) | 1573 | -1320 |
| *QR*_1_(kW) | 1452 | 1198 |
| RR2(kW) | 1.42 | 0.998 |
| *N*_F2_ | 33 | 32 |
| *N*_T2_ | 40 | 39 |
| *ID*_2_(m) | 0.777 | 0.713 |
| *QC*_2_(kW) | 1452 | 1198 |
| *QR*_2_(kW) | 1638 | 1385 |
| TAC($/y) | 7.49🞨105 | 6.46🞨105 |

**Table S9: Comparison of parameters before and after optimization**
